# Systematic analysis of Heat Shock Protein 70 (*HSP70*) gene family in radish and potential roles in stress tolerance

**DOI:** 10.1186/s12870-023-04653-6

**Published:** 2024-01-02

**Authors:** Xiaoxue Pan, Yang Zheng, Kairong Lei, Weilin Tao, Na Zhou

**Affiliations:** 1 Biotechnology Research Institute, Chongqing Academy of Agricultural Sciences/Chongqing Key Laboratory of Adversity Agriculture, Chongqing, 401329 China; 2 Vegetable and Flower Research Institute, Chongqing Academy of Agricultural Sciences, Chongqing, 401329 China; 3 Key Laboratory of Evaluation and Utilization for Special Crops Germplasm Resources in the Southwest Mountains, Ministry of Agriculture and Rural Affairs (Co-Construction By Ministry and Province), Chongqing, 401329 China

**Keywords:** *Raphanus sativus*, HSP70 family genes, Expression patterns, Abiotic stresses, *Plasmodiophora brassicae*

## Abstract

The 70 kD heat shock proteins (HSP70s) represent a class of molecular chaperones that are widely distributed in all kingdoms of life, which play important biological roles in plant growth, development, and stress resistance. However, this family has not been systematically characterized in radish (*Raphanus sativus* L.). In this study, we identified 34 RsHSP70 genes unevenly distributed within nine chromosomes of *R. sativus*. Phylogenetic and multiple sequence alignment analyses classified the RsHSP70 proteins into six distinct groups (Group A–F). The characteristics of gene structures, motif distributions, and corresponding cellular compartments were more similar in closely linked groups. Duplication analysis revealed that segmental duplication was the major driving force for the expansion of RsHSP70s in radish, particularly in Group C. Synteny analysis identified eight paralogs (Rs-Rs) in the radish genome and 19 orthologs (Rs-At) between radish and *Arabidopsis*, and 23 orthologs (Rs-Br) between radish and Chinese cabbage. RNA-seq analysis showed that the expression change of some RsHSP70s were related to responses to heat, drought, cadmium, chilling, and salt stresses and *Plasmodiophora brassicae* infection, and the expression patterns of these RsHSP70s were significantly different among 14 tissues. Furthermore, we targeted a candidate gene, *RsHSP70–23*, the product of which is localized in the cytoplasm and involved in the responses to certain abiotic stresses and *P*. *brassicae* infection. These findings provide a reference for further molecular studies to improve yield and stress tolerance of radish.

**Supplementary Information:**

The online version contains supplementary material available at 10.1186/s12870-023-04653-6.

## Background

Heat shock proteins (HSPs) are conserved stress-responsive proteins induced by adverse environmental conditions, which can be divided into six major subfamilies according to their molecular masses, i.e., HSP40, HSP60, HSP70, HSP90, HSP100, and small HSPs (sHSPs) [1]. HSP70 is the most widely studied type of HSP and is characterized by the N-terminal ATPase domain (NBD), substrate binding domain (SBD), and a variable C-terminal lid region domain [2]. Based on the subcellular localization, HSP70s in plants have been classified into four major subfamilies: those present in the cell nucleus/cytoplasm (EEVD motif), endoplasmic reticulum (HDEL motif), plastids (PEGDVIDADFTDSK motif), and mitochondria (PEAEYEEAKK motif) [3].

As molecular chaperones, the most important biological function of HSP70s is linked to acquired thermotolerance under high-temperature stress, and function as negative feedback regulators of heat shock transcription factor (HSF) activity [4–7]. In *Arabidopsis*, *cpHsc70–*1(At4g24280) is not only essential for normal plant growth but is also important for root growth from heat-stressed seeds [8]. *AtHSP70–15* affects not only the growth phenotype and leaf morphology but also the response to heat stress [9]. Mutation of the rice chloroplast OsHsp70CP1 causes chloroplast developmental defects under high-temperature conditions [10]. Plastid HSP70-2 (cpHsp70-2) involved with the temperature-dependent chalkiness of rice grains [11]. HSP70s have also been studied under high temperature stress in a variety of vegetables, such as pepper [12], pumpkin [13], potato [14], cucumber [15] and tomato [16]. In addition, HSP70 proteins in plants also improve tolerance to low-temperature, high-salinity, drought, light, flooding, and heavy metal stress in Arabidopsis, maize, rice, barley, wheat, soybean, tobacco, poplar, sugarcane, and chlamydomonas [16]. Furthermore, some HSP70s in plants are also involved in microbial pathogenesis, particularly viral infections [17]. Cytoplasmic HSP70s enhance the infection of *Nicotiana benthamiana* by tobacco mosaic virus, potato virus x, cucumber mosaic virus, and watermelon mosaic virus [18]. HSP70-depleted plants show increased susceptibility to *Pseudomonas syringae* [19], and silencing of cytosolic HSP70a in pepper results in enhanced susceptibility to *Xanthomonas campestris* infection [20]. Plants show enhanced resistance to infection by rice stripe virus (RSV) [21] through the accumulation of endoplasmic reticulum (ER)-resident HSP70s (also called binding proteins, BiPs).

Radish (*Raphanus sativus* L., 2n = 18) is an important commercial root vegetable crop that belongs to the Brassicaceae family and is sensitive to heat [22], salt [23] or heavy metal stress [24–26]. Although the HSP70 gene family is associated with different abiotic stress responses in many plant species, the genome-wide identification and functional characterization of the HSP70 family in radish have not been reported previously. In this study, 34 HSP70 family members were identified in the radish genome and divided into different classes, and the phylogenetic relationships, chromosome arrangements, gene structures, conserved motifs, and expression profiles of *RsHSP70* genes in distinct tissues or in response to various environmental stresses were also systematically analyzed. Our results provide a biological reference for elucidating the functions of HSP70 genes in radish, and will be useful for the selection of candidate genes for genetic engineering in *R*. *sativus* breeding.

## Materials and methods

### Identification of HSP70 genes

The whole-genome sequences of radish were obtained from the BRAD database (http://brassicadb.org/brad/) [27]. A Hidden Markov Model (HMM) profile containing HSP70 (Pfam: PF00012) was downloaded from the InterPro database (https://www.ebi.ac.uk/interpro/) [28], and used to confirm the candidate HSP70s in the radish genome with HMMER v3.2.1 (http://hmmer.org/) with an *E* cutoff value of 0.01. The HSP70 protein sequences of *Arabidopsis* were obtained from the TAIR database [29]. In addition, all AtHSP70s protein sequences were searched against radish protein sequences using BLASTP with an *E* cutoff value of 10^–10^. Subsequently, all of the putative radish HSP70 sequences were merged and further verified using the online SMART database (http://smart.embl-heidelberg.de/) [30]. The HSP70 protein sequences of rice, cabbage, rapeseed, and Chinese cabbage were obtained from a previous report [31]. The physicochemical parameters of each HSP70 protein were calculated using the online ExPASy server (https://www.expasy.org/resources/compute-pi-mw) [32], and the subcellular localization was analyzed using WoLF PSORT (http://www.genscript.com/psort/wolf_psort.html) [33].

### Sequence alignments and phylogenetic analysis

The full protein sequences of HSP70 from radish, *Arabidopsis*, cabbage, rapeseed, Chinese cabbage, and rice were aligned using ClustalW with the default parameters implemented in MEGA 7.0 (v7.0.21), which were used to construct the neighbor-joining (NJ) phylogenetic tree with specific parameters (model: Poisson model; gap: pairwise deletions; bootstrap replicates: 1000) [34]. The phylogenetic tree was visualized with EVOLVIEW (http://www.evolgenius.info/evolview/).

### Analyses of gene structure, and *cis*-regulatory elements

For gene structure analysis, the exon–intron organizations of individual *RsHSP70* genes were determined using the online GSDS program (http://gsds.cbi.pku.edu.cn/) [35]. The Multiple Em for Motif Elicitation (MEME v5.1.1,http://meme-suite.org/) was used to analyze the conserved motifs with the following parameters: number of repetitions: any; maximum number of motifs: 20; optimum motif widths: 10–100 amino acid residues [36]. The exon–intron organizations and conserved motifs were visualized with TBtools (v1.108) [37]. The online PlantCARE database (http://bioinformatics.psb.ugent.be/webtools/plantcare/html/) was used to determine the putative *cis*-acting regulatory elements, as described previously [38].

### Chromosomal location and identification of orthologous and paralogous genes

The radish genome was searched for all *RsHSP70* gene loci and the corresponding location was visualized with Circos software (v 0.69) [39]. Duplicated *RsHSP70* gene pairs were identified using MCScanX with the default settings [40]. The nonsynonymous replacement (Ka) and synonymous replacement (Ks) rates were estimated using Ka/Ks Calculator 2.0 implemented in TBtools (v1.108) [37], The formula T = Ks/2λ (λ = 6.96 × 10^–9^) was used to estimate the divergence time of duplication events [41]. OrthoMCL(v6.18) (http://orthomcl.org/orthomcl/app) was used to investigate potential orthologous and paralogous HSP70 genes in radish, *Arabidopsis*, and Chinese cabbage with the standard settings, and visualized with Circos (v 0.69) [39].

### Expression analysis of RsHSP70 genes in various tissues or under different stresses

To examine the potential functions of *RsHSP70* genes in different tissues of radish, the raw RNA-seq data of 14 tissues were acquired from a previous report [42] (Table S 1). These data represented five tissues (leaf, root tip, cortex, cambium, and xylem) and five stages (7, 14, 20, 40, and 60 days after sowing, DAS). To investigate whether *RsHSP70* genes play important roles in various stress responses in radish, data from seedlings exposed to heat, drought, cadmium (Cd), chilling, salt stress were obtained from the NCBI SRA database (SUB13505845) and *P.brassicae* infection were obtained from the CNCB database (https://ngdc.cncb.ac.cn/) with the number CRA004024. The expression level of each gene presented in transcripts fragments per kilobase of exon model per million mapped fragments (FPKM) was reanalyzed as described previously [43]. Differentially expressed genes (DEGs) were selected with cutoff values of |log2fold-change|> 1 and *p* < 0.05. Heat maps were generated in R (v3.6.3) with log2-transformed FPKM values after the addition of a pseudocount of 0.01.

### Plant material and treatment

Two inbred radish lines with contrasting resistance to *P. brassicae*, WR1150 and 1116 T, (resistant and susceptible, respectively) obtained from the Institute of Vegetables and Flowers, Chongqing Academy of Agricultural Sciences, China, were used. The seeds of the two lines were surface-sterilized, sown in trays containing a 3:1 mixture of nutrient soil and sand, and grown under conditions of 25 °C/20 °C (day/night) with a 16 h photoperiod. The pathogen used in this study was obtained from the natural clubroot nursery at Wulong, Chongqing, China, where *P. brassicae* race 4 is the dominant clubroot pathogen. *P. brassicae* suspensions were prepared as described previously [44], and inoculate we prepared by diluting the suspension to a resting spore concentration of 1 × 10^8^/mL. Ten similar seedlings with 20-day leaves were individually injected at the bottom of the stem with 5 mL spore suspension. Total RNA was isolated from roots collected at 0, 4, 7, 14, 21, and 28 days post inoculation (dpi) of* P*. *brassicae* for qRT-PCR analysis.

To analyze abiotic stress, five similar seedlings of 1116 T with 20-day leaves were treated with CdCl_2_·2.5H_2_O (200 mg/L), mannitol (300 mM), or NaCl (200 mM) for 48 h [23, 24], or by chilling (4 °C) or heating (40 °C) for 24 h [22]. Leaves were collected at each time point and used to isolate total RNA.

### RNA isolation and qRT-PCR analysis

Total RNA was isolated from 20-day leaves using an RNAprep pure Plant Kit (Catalog no.dp432; Tiangen Biotech Co., Ltd., Beijing, China), and quantified using the NanoDrop ND-1000 (Termo Scientifc, Waltham, MA, USA).The first-stand cDNA was synthesized using a HiScript III 1st Strand cDNA Synthesis Kit (+ gDNA wiper) (Catalog no.R312–01; Vazyme Biotech Co., Ltd., Nanjing, China). ChamQ Universal SYBR qPCR Master Mix (Catalog no.Q711–02; Vazyme Biotech) was used for qRT-PCR on a BIO-RADCFX96 Real Time System (Bio-Rad Laboratories, Hercules, CA, USA). Three independent biological replicates were used for each sample. The transcript levels were calculated using the ΔΔC_T_ method with normalization relative to the level of *RsActin* [45]. All gene-specific primers used in this study are presented in Table S 2.

### Determination of subcellular localization

For transient expression, the coding sequence of RsHSP70–23 was inserted into pAN580 to generate GFP fusion proteins. The plasmids pAN580-GFP-35Spro:GFP and pAN580-GFP-35Spro:RsHSP70–23-GFP were transformed into Arabidopsis protoplasts. The GFP signals were detected by confocal laser microscopy (Carl Zeiss, Oberkochen, Germany).

### Yeast constructs, tolerance assay and growth curve

The coding sequence of *RsHSP70–23* was inserted into pRS-416-GFP, and the recombinant vector and the empty pRS-416-GFP vector (control) were transformed into the wild-type strain JRY472 and allowed to grow on SD-Ura plates. Positive recombinant transformants cultured in SD-Ura medium were diluted until OD_600_ = 0.1. Then the cell culture was diluted ten fold and treated with 75 µM Cd, 1 M NaCl, and 2 M mannitol, and incubated at 30 °C for 3 days. Cold and heat stress were applied at 4 °C and 37 °C for 2 days before transfer to 30 °C for 1 or more days [46]. Then, the phenotypes of the yeast cells were photographed, and the experiment was repeated three times. RsHSP70-23 overexpressing yeast cells were grown in liquid SD-Ura medium at 4℃, 30℃ and 37℃, respectively, and with 1 M NaCl were also grown at 30℃, and were diluted until OD600 = 0.1, OD600 is recorded every 2 h to prepare cell growth curve [46].

## Results

### Identification of *RsHSP70* genes in radish genome

Through a combination of BLASTP and HMM profile analysis, 34 putative *RsHSP70* genes were identified in the “Xiangyabai” genome and renamed according to their relative linear order on each chromosome (Table 1). Among them, the lengths of predicted RsHSP70 protein sequences varied from 112 (RsHSP70–32) to 1335 (RsHSP70–4) amino acids with molecular weights (MWs) ranging from 12.21 to 144.26 kDa. The theoretical isoelectric points (pIs) of most predicted RsHSP70 proteins were < 7, with the exceptions of RsHSP70–4, RsHSP70–29, RsHSP70–30, and RsHSP70–32. In addition, WoLF PSORT online analysis predicted that within the 34 RsHSP70 proteins, 19 radish HSP70 proteins were localized to the cytosol/nucleus, 7 to the chloroplast, 4 to the mitochondria, and 4 to the ER (Table 1).

**Table 1:** The general information and sequence characterization of 34 *RsHSP70* genes

S.N	Gene^a^	Locus^b^	Location^c^	ORF(bp)^d^	Exon^e^	Protein^f^			Subcellular location^g^
						**Size (aa)**	**MW(d)**	**pI**	
1	RsHSP70-1	Rsa10038515	R01:6,368,319–6,370,518	1692	1	563	60,475.48	5.27	cyto: 8, chlo: 5
2	RsHSP70-2	Rsa10012256	R01:32,783,050–32786226	1908	4	635	68,894.74	4.73	cyto: 9, cysk: 3, chlo: 1
3	RsHSP70-3	Rsa10034690	R01:48,749,418–48,750,437	1023	1	340	36,918.8	4.49	cyto: 8, nucl: 2.5, cysk_nucl: 2, chlo: 1, mito: 1
4	RsHSP70-4	Rsa10021747	R02:6,150,715–6157881	4008	11	1335	144,259.7	8.32	chlo: 8, mito: 2, vacu: 2, plas: 1
5	RsHSP70-5	Rsa10021746	R02:6,158,811–6,162,178	1695	8	564	60,435.85	4.4	cyto: 10, mito: 2, pero: 2
6	RsHSP70-6	Rsa10018910	R02:9,695,473–9,699,355	2700	14	899	99,811.07	5.47	chlo: 4, nucl: 3, plas: 3, cyto: 1, vacu: 1, E.R.: 1
7	RsHSP70-7	Rsa10024964	R02:12,928,642–12,933,512	2052	6	683	73,014.51	5.58	mito: 10, chlo: 4
8	RsHSP70-8	Rsa10030573	R02:37,598,054–37599295	1245	1	414	47,290.77	5.16	cyto: 5, nucl: 5, plas: 1, cysk: 1, golg: 1
9	RsHSP70-9	Rsa10036337	R03:1,667,438–1,673,472	2052	5	683	72,750.21	5.34	mito: 12, chlo: 2
10	RsHSP70-10	Rsa10032304	R03:8,248,787–8,251,621	2046	6	681	72,578.1	4.83	E.R.: 7, chlo: 3, cyto: 2, extr: 1
11	RsHSP70-11	Rsa10036631	R04:4,165,044–4167982	2139	8	712	76,140.85	5.13	chlo: 14
12	RsHSP70-12	Rsa10025332	R04:6,821,393–6,825,331	2691	14	896	99,731.31	6.07	E.R.: 6, chlo: 4, vacu: 2, cyto: 1
13	RsHSP70-13	Rsa10005413	R04:49,330,589–49334805	2445	9	814	90,108.18	4.89	cyto: 9, nucl: 3, chlo: 2
14	RsHSP70-14	Rsa10042765	R05:2,174,643–2,176,040	717	1	238	25,867.96	4.46	cyto: 6, mito: 5, chlo: 1, nucl: 1
15	RsHSP70-15	Rsa10041785	R05:7,596,122–7,598,778	1956	2	651	71,190.63	4.78	cyto: 8, cysk: 4, chlo: 1
16	RsHSP70-16	Rsa10019803	R05:16,754,020–16756483	1896	5	631	69,998.34	4.68	cyto: 13
17	RsHSP70-17	Rsa10010269	R05:27,677,825–27,680,504	2067	6	688	72,115.42	4.72	E.R.: 7, chlo: 3, cyto: 2, extr: 1
18	RsHSP70-18	Rsa10009146	R06:1,006,847–1010066	2454	9	817	90,061.33	4.92	cyto: 9, chlo: 3, nucl: 2
19	RsHSP70-19	Rsa10010766	R06:4,867,783–4,868,904	1125	1	374	42,038.14	5.4	nucl: 6, cyto: 6, mito: 1
20	RsHSP70-20	Rsa10039994	R06:16,654,115–16,655,488	1377	1	458	50,374.95	4.72	cyto: 8, chlo:6
21	RsHSP70-21	Rsa10038047	R06:20,575,035–20578239	1947	1	648	71,048.53	5.33	cyto: 13
22	RsHSP70-22	Rsa10037733	R06:25,576,889–25,580,215	2271	9	756	83,993.89	5.92	nucl: 10, chlo: 1, cyto: 1, vacu: 1
23	RsHSP70-23	Rsa10031033	R07:2,236,711–2,239,183	1959	5	652	70,528.96	4.79	E.R.:5, chlo: 4, cyto.: 2, extr: 2
24	RsHSP70-24	Rsa10023626	R07:12,631,430–12632851	1425	1	474	52,005.73	4.7	cyto: 7, chlo: 6
25	RsHSP70-25	Rsa10007904	R07:25,365,226–25,367,405	1944	2	647	70,846.34	4.81	cyto: 9, cysk: 3, chlo: 1
26	RsHSP70-26	Rsa10010304	R08:17,739,581–17,741,005	1428	1	475	52,154.89	4.67	cyto: 7, chlo: 6
27	RsHSP70-27	Rsa10026855	R08:18,902,782–18903463	549	2	182	20,604.26	6.81	mito: 7, cyto: 6.5, nucl: 4
28	RsHSP70-28	Rsa10026857	R08:18,911,557–18,914,418	2043	8	680	72,260.14	4.85	chlo: 14
29	RsHSP70-29	Rsa10026985	R08:19,773,591–19,775,985	1986	6	661	65,334.75	8.85	mito: 12, chlo: 2
30	RsHSP70-30	Rsa10026987	R08:19,779,354–19,784,550	3510	11	1169	125,986.3	7.44	chlo: 8, mito: 5
31	RsHSP70-31	Rsa10040958	R08:24,052,015–24054747	1968	1	655	71,607.85	5.05	cyto: 11, chlo: 2
32	RsHSP70-32	Rsa10023846	R09:27,216,803–27217385	339	3	112	12,208.96	8.21	chlo: 11, cyto: 1, plas: 1
33	RsHSP70-33	Rsa10015012	R09:33,292,486–33,293,068	414	3	137	15,093.08	5.69	chlo: 7, mito: 3, nucl: 2, cyto: 1
34	RsHSP70-34	Rsa10002306	Scaffold326:1636–3069	1437	1	478	52,452.2	4.67	cyto: 7, chlo: 5, E.R.: 1

^a^ Systematic designation given to radish *HSP70s* in this study

^b^ Locus identity number of *RsHSP70s* assigned by BRAD database (http://brassicadb.org/brad/)

^c^ Chromosomal localization of radish HSP70 genes

^d^ Length of the open reading frame

^e^ Number of extrons obtained from GSDS by comparing sequences between transcript and genome (Gene Structure Display Server; http://gsds.cbi.pku.edu.cn/)

^f^ Protein characterization of RsHSP70s obtained from EXPASY server (http://web.expasy.org/protparam/)

^g^ Subcellular localization of each HSP70 protein obtained from WoLF PSORT (https://wolfpsort.hgc.jp/). chlo, chloroplast; cyto, cytosol; nucl, nucleus; E.R., endoplasmic reticulum; mito, mitocondria; plas, plasma membrane; extr, extracelular; cysk, cytoskeleton; pero, peroxisome; vacu, vacuolar membrane; golg, Golgi S.N., serial number; ORF, open reading frame; bp, base pair; aa, amino acids; MW, molecular weight; pI, isoelectric point

### Six groups defined among the HSP70 genes of six species

**Fig. 1:**
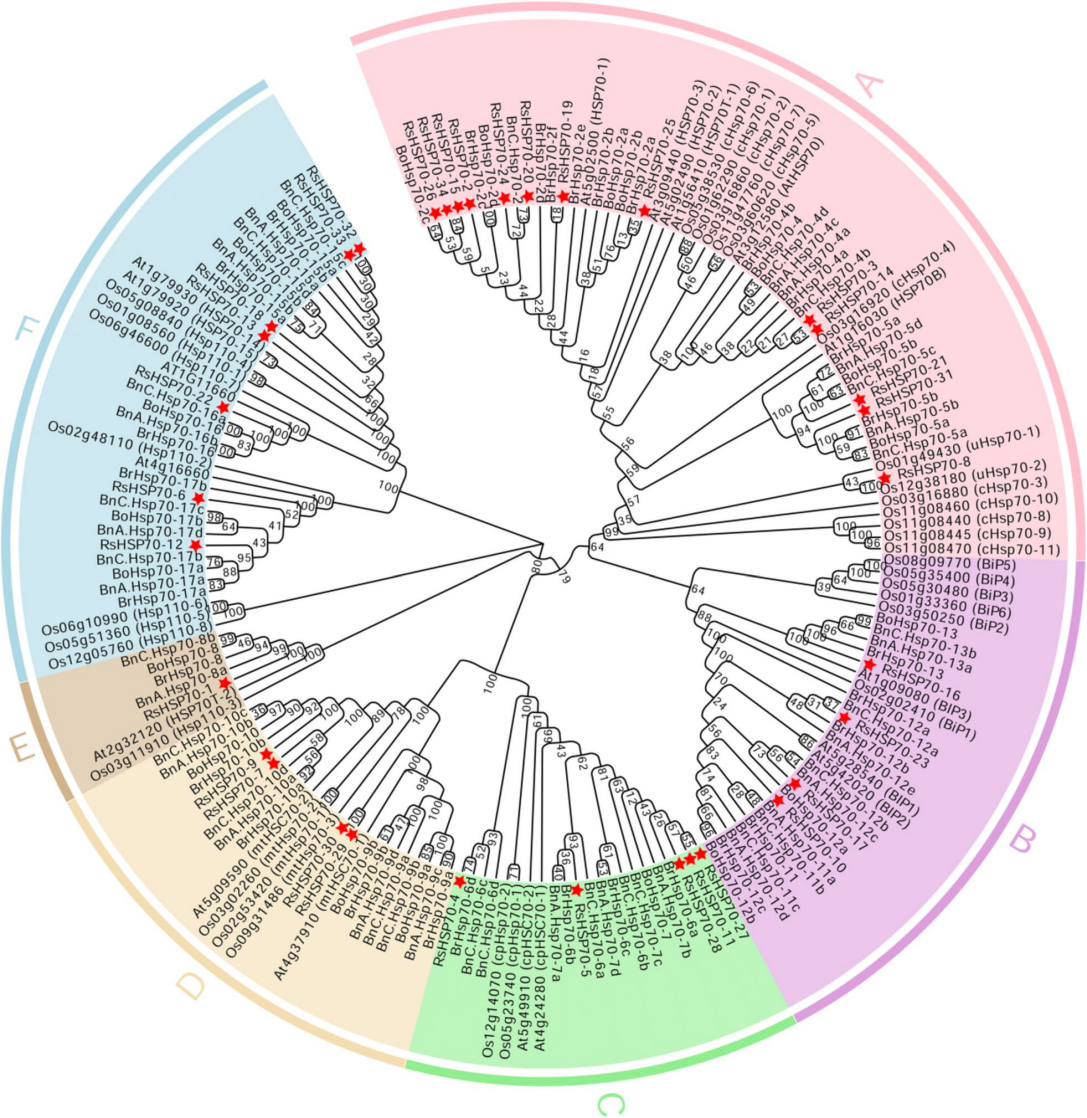
Phylogenetic relationships of radish, rapeseed, Chinese cabbage, cabbage, rice, and Arabidopsis HSP70 proteins. The tree was divided into six subgroups, marked by different color backgrounds

To investigate the phylogenetic relationships of these HSP70s, the full-length amino acid sequences of RsHSP70s (34), BnHSP70s (*Brassica napus*, 47), BrHSP70s (*Brassica rapa*, 29), BoHSP70s (*Brassica oleracea*, 20), OsHSP70s (*Oryza sativa*, 32), and AtHSP70s (*Arabidopsis thaliana*, 18) were used to construct a neighbor-joining (NJ) phylogenetic tree; they clustered into six distinct groups (Groups A–F) (Fig. 1). Among them, Group A was the most abundant subfamily containing 58 members, consisting of 13 RsHSP70 members that were predicted to be localized in the cytoplasm/nucleus (Table 1). Groups B, C, and D consisted of four, five, and four members from radish, which were predicted to be located in the ER, chloroplast, and mitochondria, respectively. These results suggest that the closely related HSP70s are usually located in the same subcellular structure. In total, 38 members belonging to the Hsp110/SSE subfamily were classified into Group F, which also contained 7 RsHSP70 members. Group E only had one RsHSP70, which was suggested to be a truncated gene based on comparative analysis with its *Arabidopsis* counterpart [47]. Each group contained proteins from both monocotyledonous and dicotyledonous plants, indicating that the main characteristics of HSP70 proteins in plants emerged before the separation of these two lineages.

### Characterization of RsHSP70 proteins and distributions of conserved motifs and gene structures

**Fig. 2:**
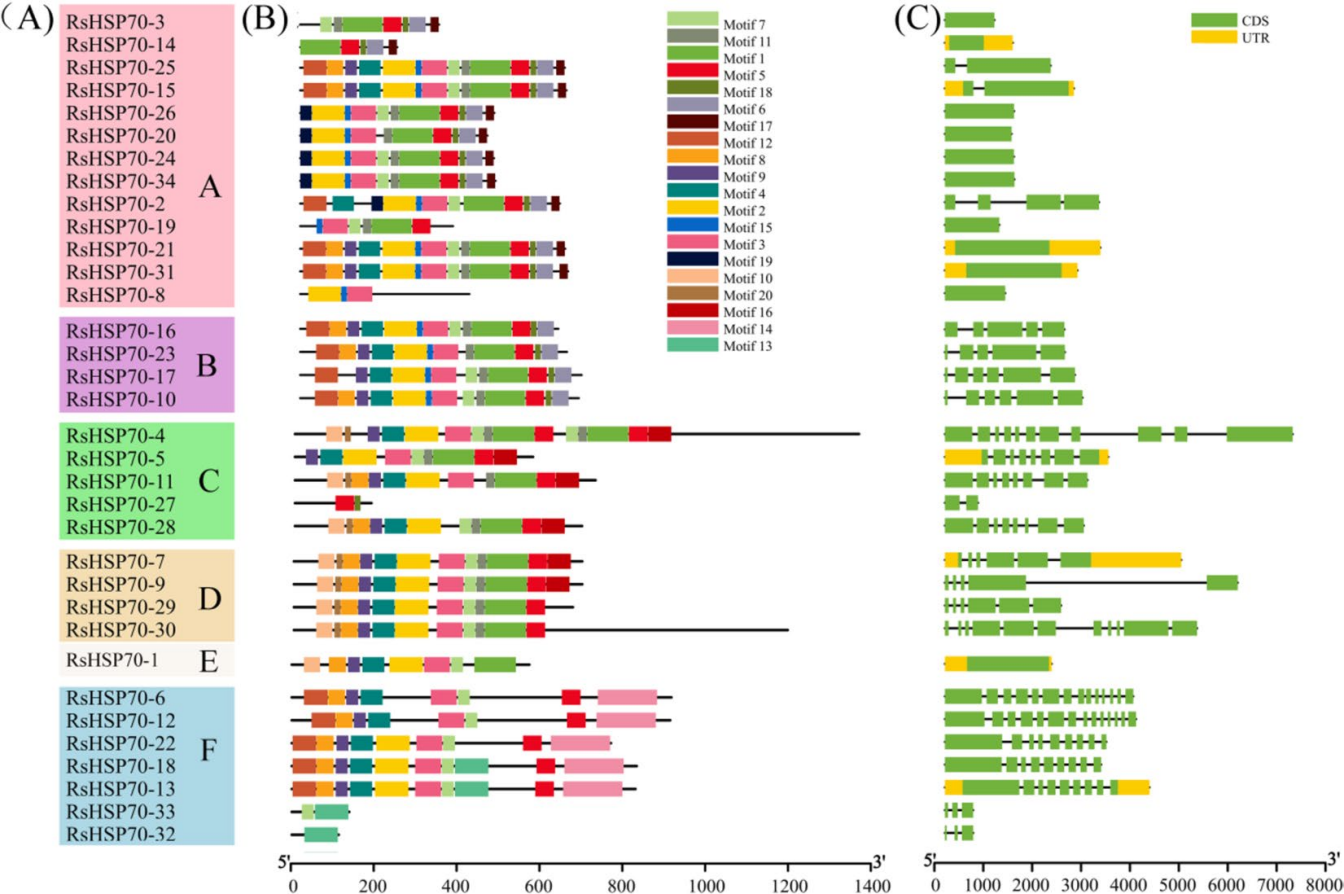
Phylogenetic relationship, gene structures and conserved motifs of RsHSP70s. **A** Phylogenetic tree of 34 RsHSP70s proteins. **B** Conserved motifs of RsHSP70s proteins. Each colored box represents a specific motif in the protein identified using the MEME motif search tool. **C** Exons and introns were indicated by green rectangles and red lines respectively

Sequence alignments revealed that most HSP70 family proteins in radish included three distinct domains. The highly conserved NBD domain possessed three HSP70 signature sequences, the SBD domain was also conserved, while the C-terminal domain was highly variable (Figure S1). However, RsHSP70–14 and RsHSP70–27 did not include the NBD domain, and RsHSP70–32 and RsHSP70–33 lacked the C-terminal domain. All 34 RsHSP70s clustered into six groups in phylogenetic analysis (Fig. 2A). The majority (11/13, 84.6%) of RsHSP70s in Group A possessed a conserved retention signal “EEVD” sequence at the C-terminus, and were localized in the cytoplasm. Half (2/4) of those in Group B were localized in the ER and 60% (3/5) of those in Group C were localized in the chloroplasts, and possessed the conserved C-terminal signature sequences “HDEL” and “DVIDADFTDSK,” respectively. In addition, the three RsHSP70s in Group D possessed the conserved signature sequence “PEAEYEEAKK” in the C-terminus, and were localized in the mitochondria (Figure S1).

Twenty conserved motifs were identified in radish HSP70 proteins using the MEME motif search tool (Fig. 2B and Table S 3). Motifs 2 (24/34), 3 (26/34), and 12/10 (22/34) were found in the RsHSP70 family, corresponding to signature sequences. Motifs 1 (23/34), 5 (29/34), and 7 (25/34) were included in the SBD domain. Similar compositions, orders, and numbers of motifs were found in the groups localized to the mitochondria and chloroplasts. However, some subfamilies also included several specific motifs. For example, motifs 13 and 14 were exclusively present in the HSP110/SSE subfamily, which lacked motif 1; motif 19 was only found in those localized to the cytosol; and motifs 16 and 20 were absent in those localized to the cytosol or ER.

The online GSDS tool was used to identify the exon–intron organization in the coding sequences shared among the RsHSP70s (Fig. 2C). The number of exons in RsHSP70s varied from 1 to 14. Most closely related members that clustered together shared similar exon–intron organization and exon length. For example, the two members of the HSP110/SSE subfamily have 13 introns and 14 exons, and are nearly 2700 bp in length, while 84.6% of cytosolic HSP70s had no introns. These results indicate a diversity of exon–intron organization in the radish HSP70 family.

### Gene duplication and synteny analysis of RsHSP70s

**Fig. 3:**
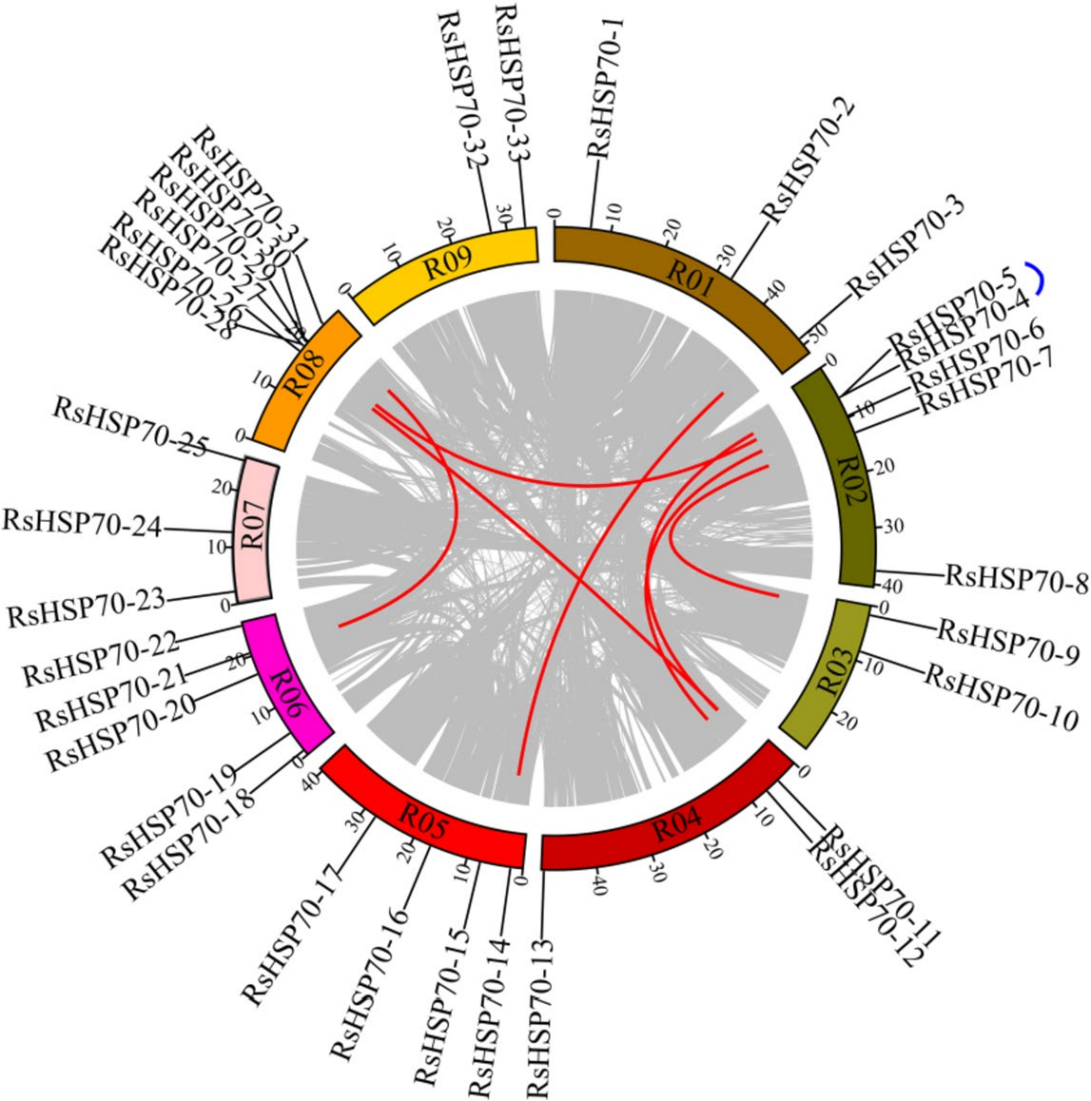
Chromosomal localization and gene duplication events of RsHSP70 genes. Respective chromosome numbers are indicated at the top of each bar. Tandem duplicated genes are marked on a grey background. Segmental duplicated genes are shown by black line

Chromosome location analysis showed that in addition to RsHSP70–34 the remaining 33 RsHSP70 genes were irregularly distributed on the nine chromosomes in radish (Fig. 3). Chromosome 8 had the greatest number of HSP70 genes (*n* = 6), followed by five genes on each of chromosomes 2 and 6, four genes on chromosomes 4, three genes on each of chromosomes 1, 7 and 8, and only two on each of chromosomes 3 and 9.

To investigate the forces driving gene family expansion, tandem and segmental duplication patterns were examined. Among the 34 *RsHSP70* genes, 35.29% (12/34) arose from duplication events, including one pair derived from tandem duplication and 11 genes derived from segmental duplications (Fig. 3). The tandem duplicated genes were *RsHSP70–4* and *RsHSP70–5* located on chromosome 2. The 11 segmental duplicated *RsHSP70* genes were classified into five groups. One group consisted of three genes, *RsHSP70–4*, *RsHSP70–11*, and *RsHSP70–27*, located on chromosomes 2, 4, and 8, respectively. The other four groups consisted of pairs of genes: *RsHSP70–3* and *RsHSP70–14*, *RsHSP70–6* and *RsHSP70–12*, *RsHSP70–7* and *RsHSP70–9*, and *RsHSP70–21* and *RsHSP70–31*. It was noteworthy that *RsHSP70–4* underwent tandem duplication before segmental duplication (Table 2). The Ka/Ks ratio varied from 0.014 to 0.388, indicating that these genes were subject to purifying selection. The segmental duplication occurred at 2.49–7.58 million years ago (Mya), while tandem duplication occurred at 3.32 Mya.

**Table 2:** Ka-Ks calculation for each pair of HSP70 in radish

Paralog pairs	S-sites	N-sites	Ka	Ks	Ka/Ks	Selection pressure	Duplication type	Duplication time (Mya)
RsHSP70-11/RsHSP70-27	117.17	413.83	0.24	1.05	0.23	Purifying selection	Segmental	7.58
RsHSP70-21/RsHSP70-31	444.33	1496.67	0.03	0.67	0.04	Purifying selection	Segmental	4.82
RsHSP70-3/RsHSP70-14	151.92	556.08	0.01	0.89	0.01	Purifying selection	Segmental	6.37
RsHSP70-4/RsHSP70-11	487.42	1558.58	0.10	0.65	0.16	Purifying selection	Segmental	4.69
RsHSP70-4/RsHSP70-27	103.92	382.08	0.26	0.68	0.39	Purifying selection	Segmental	4.86
RsHSP70-6/RsHSP70-12	603.67	2009.33	0.03	0.37	0.10	Purifying selection	Segmental	2.62
RsHSP70-7/RsHSP70-9	488.83	1548.17	0.02	0.35	0.05	Purifying selection	Segmental	2.49
RsHSP70-4/RsHSP70-5	382.42	1243.58	0.10	0.46	0.22	Purifying selection	Tandem	3.32

*S-sites* Number of synonymous sites, *N-sites* Number of non-synonymous sites, *Ka* Non-synonymous substitution rate, *Ks* Synonymous substitution, *Mya* Million years ago

**Fig. 4:**
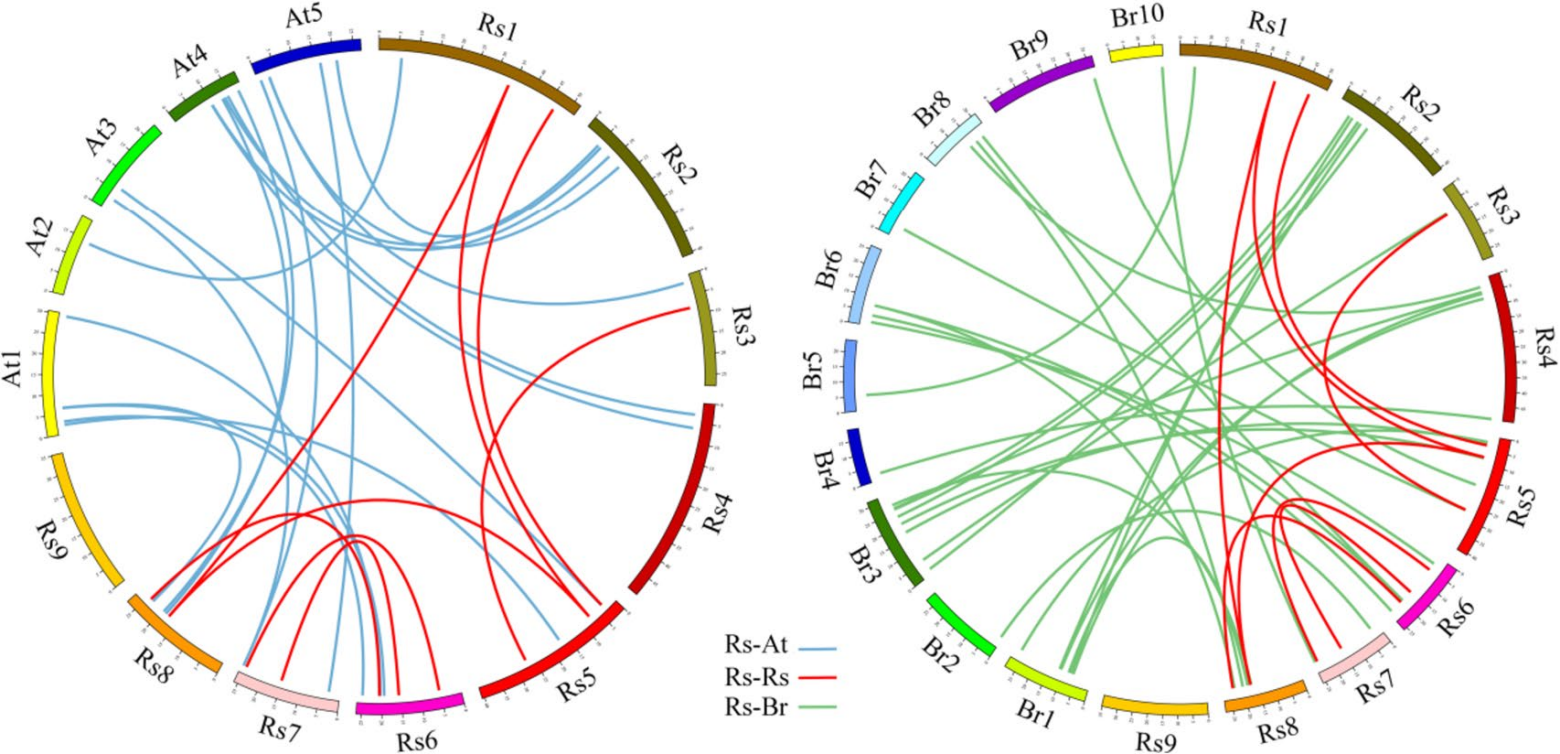
The syntenic relationship of HSP70 genes between radish and Arabidopsis, radish and Chinese cabbage. Lines in red indicate radish paralogous genes pairs, both blue line and green line indicate orthologous gene pairs

Synteny analysis showed the presence of 8 paralogs (Rs-Rs) in the radish genome, 19 orthologs (Rs-At) between radish and *Arabidopsis*, and 23 orthologs (Rs-Br) between radish and Chinese cabbage (Fig. 4 and Table S 4). Between radish and *Arabidopsis*, *AT4G24280* (*cpHSC70–1*) has three orthologous genes (*RsHSP70–4*, *RsHSP70–11*, and *RsHSP70–27*), *AT1G16030* (*HSP70B*), *AT4G16660*, and *AT5G09590* (*mtHSC70–2*) have two orthologous genes, and six *AtHSP70s* had only one orthologous gene, e.g., *AT5G42020(BIP2)/RsHSP70–23*, *AT1G09080(BIP3)/RsHSP70–16*, and *AT2G32120(HSP70T-2)/RsHSP70–1*, suggesting that there have been gene losses in many orthologous groups. It is noteworthy that 13 *RsHSP70* genes (*RsHSP70–1/-4/-6/-7/-9/-11/-12/-16/-21/-22/-27/-29/-31*) were shared between the orthologs identified in Rs-At and Rs-Br. These results provide a theoretical foundation for further exploration of *RsHSP70* gene functions.

### Expression patterns of radish HSP70s in various tissues

**Fig. 5:**
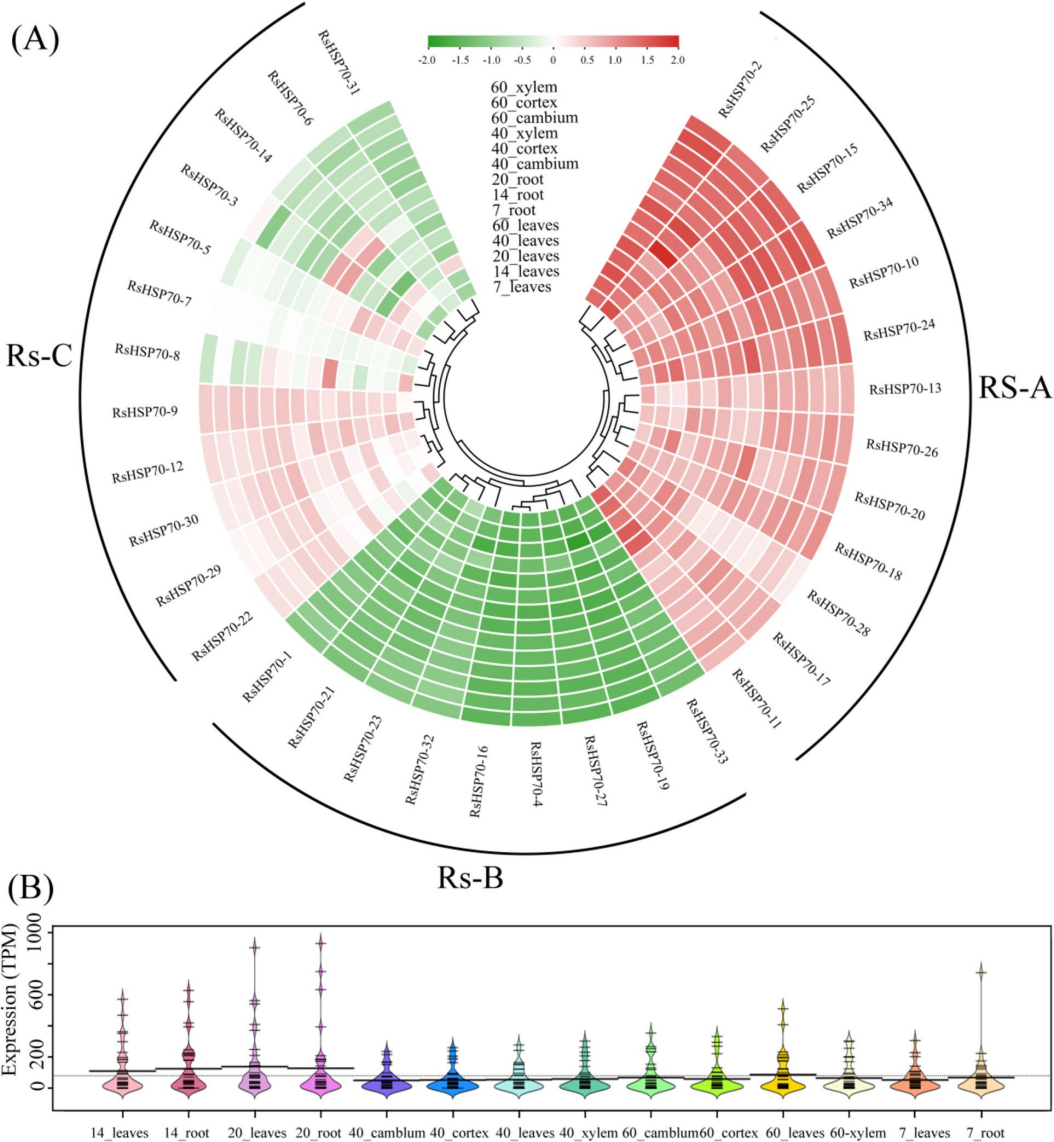
Expression profiles of RsHSP70 genes in different tissues. **A** The normalized expression levels of the hierarchical clustering of 34 RsHSP70 genes in 14 tissues. The relative expression levels corresponding to the log2-transformed TPM values after the addition of a pseudocount of 0.01 are shown. The scale represents the relative signal intensity of the TPM values. **B** The expression values of all the RsHSP70 genes in each tissue

Based on RNA-seq data, we compared the expression profiles of all *RsHSP70* genes in 14 tissues (Fig. 5A and Table S 5). The expression patterns were divided into three major groups. In subgroup Rs-A, all of the genes maintained a relatively high transcription level in all tissues examined, particularly *RsHSP70–2*, *RsHSP70–25*, and *RsHSP70–34* (TPM > 100). Nine *RsHSP70s* (*RsHSP70–1*, -*4*, -*16*, -*19*, -*21*, -*23*, -*27*, -*32*, and -*33*) in subgroup Rs-B showed low or no expression in all of the tissues examined (TPM < 5). Twelve genes in subgroup Rs-C showed differential expression between different tissues or developmental stages, while some genes maintained higher expression in specific organs, such as *RsHSP70–5* showing selective high-level expression in leaves, and *RsHSP70–31* showing higher expression in 20-day leaves compared to all other tissues examined. Most RsHSP70 genes showed higher expression levels in 20-day leaves and roots (Fig. 5B), suggesting that they may play vital roles in seedling development.

Interestingly, three pairs of paralogs (*RsHSP70–6*/*RsHSP70–12*, *RsHSP70–7*/*RsHSP70–9*, and *RsHSP70–21*/*RsHSP70–31*) showed different expression levels in different tissues, suggesting that duplicated genes may acquire new functions and increase adaptation to stressful environmental conditions. Moreover, five pairs of paralogs (*RsHSP70–2*/*RsHSP70–34*, *RsHSP70–3*/*RsHSP70–14*, *RsHSP70–10*/*RsHSP70–17*, *RsHSP70–29*/*RsHSP70–30*, and *RsHSP70–32*/*RsHSP70–33*) exhibited similar expression levels, possibly indicating subfunctionalization in the adaptation to abiotic stresses (Fig. 5A).

### Expression profiles of radish HSP70 genes under abiotic stress

**Fig. 6:**
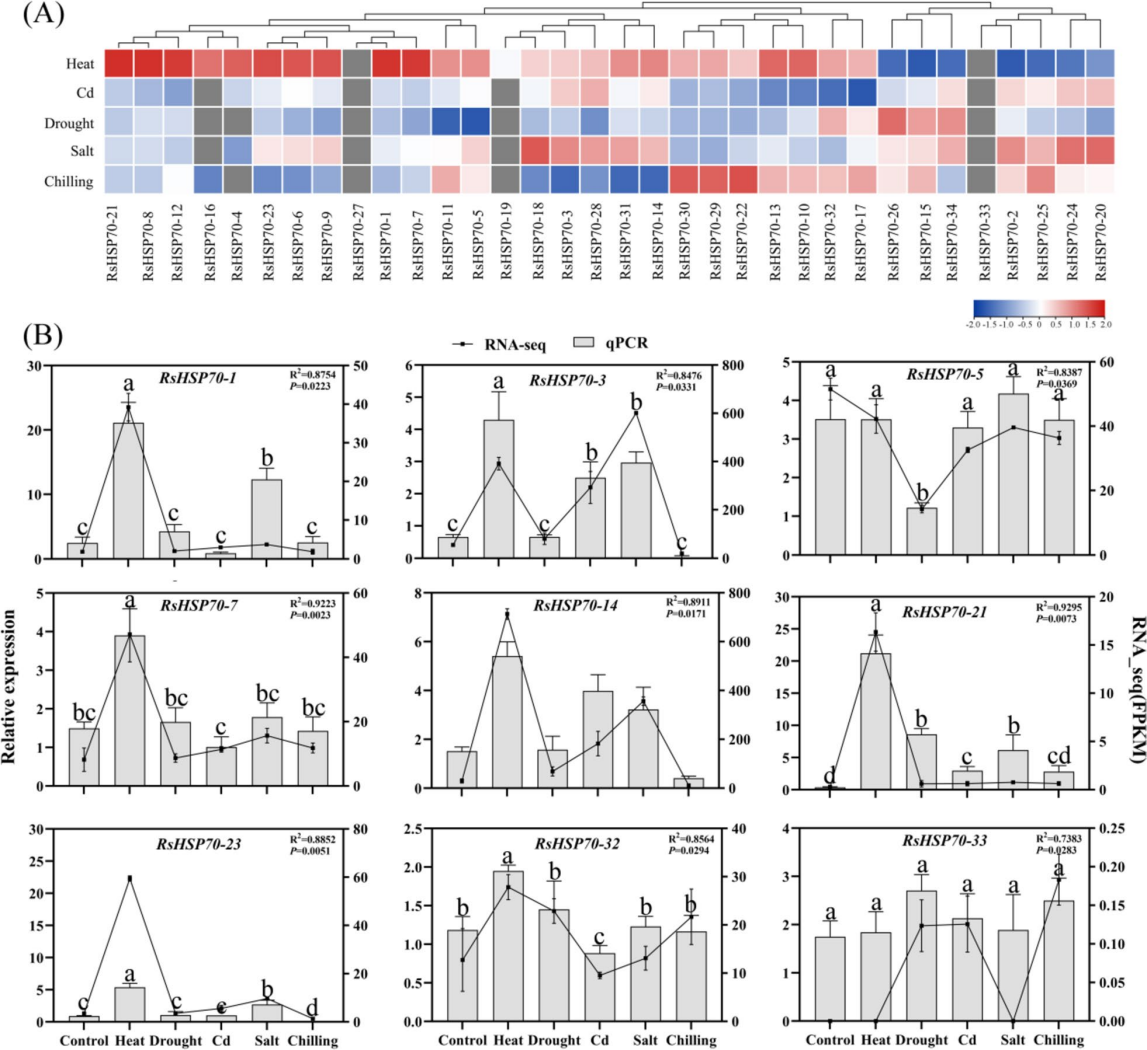
Expression profiles of RsHSP70 genes in response to abiotic stress treatments. **A** Heatmap of differential expression of RsHSP70 genes in response to heat, drought, cd, salt and chilling stress. The color scale of heatmap is based on the log2-transformed FPKM values after the addition of a pseudocount of 0.01 between experimental and control tissues are shown. **B** Expression profiles of selected genes under heat, drought, cd, salt and chilling stresses from RNA-seq data and qPCR analysis. Correlation (R.^2^ = 0.7383–0.9295, *P* < 0.05) between RNA-seq data and qPCR results. Each bar shows the mean ± SE of the triplicate assay. Different letters indicate significant differences among treatments (*P* < 0.05, ANOVA)

RNA-seq analyses showed that 25 genes were differentially expressed after exposure to heat, Cd, drought, high salinity, and chilling stresses (Fig. 6A and Table S 6). Seven *RsHSP70s* (*RsHSP70–3*, *RsHSP70–4*, *RsHSP70–8*, *RsHSP70–14*, *RsHSP70–20*, *RsHSP70–21*, and *RsHSP70–31*) were significantly upregulated following treatment with Cd and NaCl. The expression levels of three *RsHSP70* genes changed only under conditions of drought stress (*RsHSP70–5*, *RsHSP70–26*, and *RsHSP70–34*). Under low-temperature conditions, the expression levels of *RsHSP70–3*, *RsHSP70–14*, *RsHSP70–23*, and *RsHSP70–28* were significantly downregulated. Sixteen *RsHSP70* genes were consistently upregulated under conditions of heat stress, including *RsHSP70–1*, *RsHSP70–4*, *RsHSP70–8*, *RsHSP70–14*, *RsHSP70–16*, *RsHSP70–19*, *RsHSP70–21*, *RsHSP70–23*, and *RsHSP70–31* (4- to eightfold increases). We predicted the presence of several *cis*-elements that were responsive to environmental stressors (drought, low temperature, stress defense mechanism, and wounding) and hormones (abscisic acid [ABA], auxin [indole-3-acetic acid, IAA], gibberellin [GA3], methyl jasmonate [MeJA], and salicylic acid [SA]) in the promoter regions of *RsHSP70s* (Table S 7). Using the PlantPAN3 online database, we found that 31 *RsHSP70s* contained the heat shock factor (HSF1)-binding element (HSE) consisting of two main subunits (5′NGAAN3′ and 5′NTTCN3′)(Table S 8). These results suggest that most *RsHSP70* genes are induced and/or repressed by abiotic stress. To assess the accuracy of the RNA-seq data, nine *RsHSP70* genes from different groups were examined by qRT-PCR analysis under conditions of heat, salt, cold, drought, and Cd stress. The gene expression patterns obtained by qRT-PCR were generally consistent with the results of RNA-seq analyses (Fig. 6B).

### Potential roles of HSP70 genes in radish root with* P. brassicae* infection

**Fig. 7:**
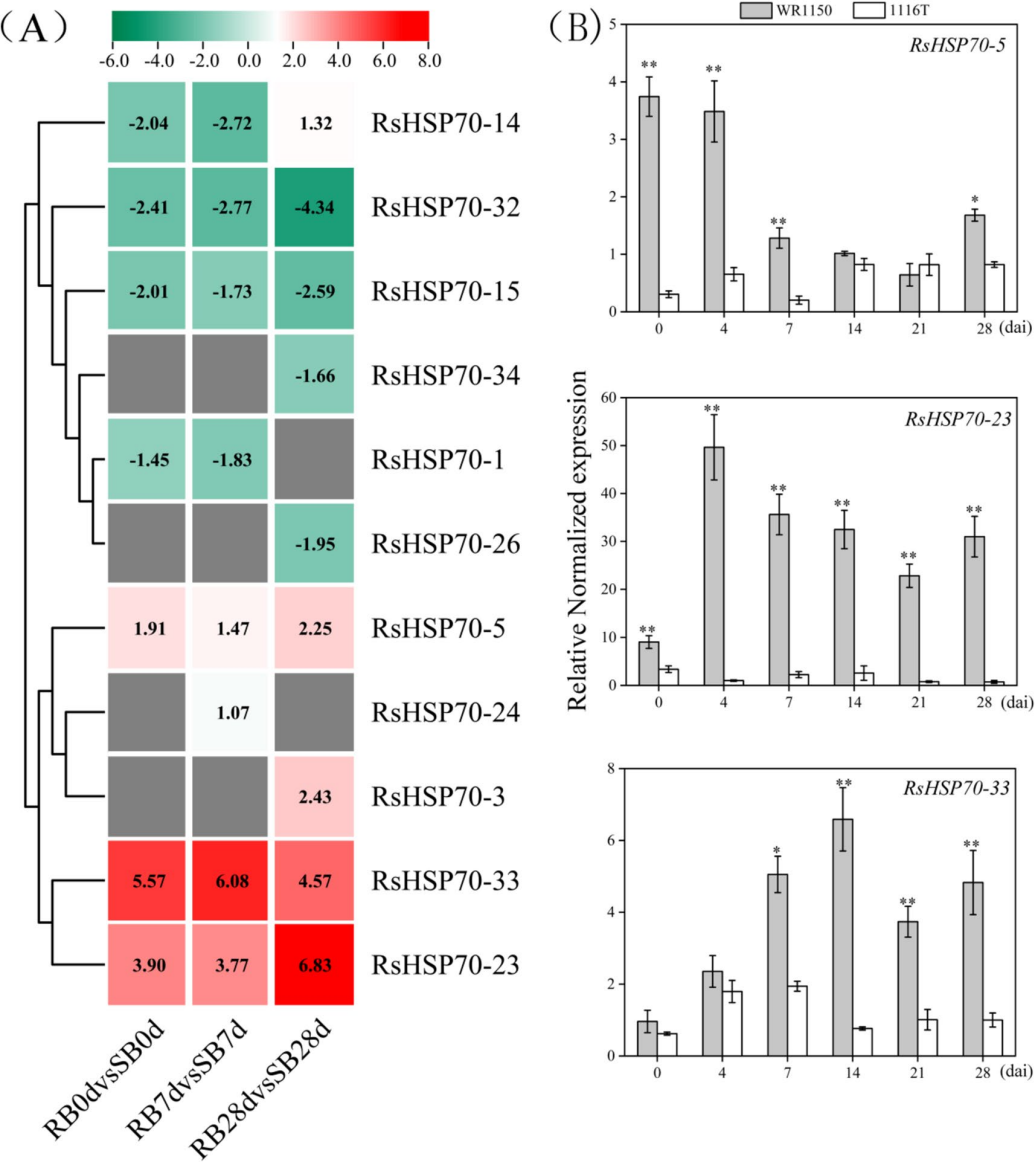
Expression profiles of RsHSP70 genes in response to *P.brassicae* infection. **A** Heatmap of differential expression of RsHSP70 genes in response to *P.brassicae* infection in RB and SB at 0, 7 and 28 dpi. The color scale of heatmap is based on the log2Foldchange values between RB and SB are shown. **B** RT-PCR analysis of selected genes under *P. brassicae* infection in WR1150 and 1116 T at 0, 4, 7, 14, 21 and 28 dpi. Error bars indicate the SD for three independent replicates. * and ** indicate a signifcant diference between WR1150 and 1116 T at *P* < 0.05 and *P* < 0.01 levels, respectively (two tailed T-test)

Most *HSP70* genes in *Arabidopsis* are upregulated in response to biotic stressors [17]. To explore the roles of *RsHSP70s* genes responsive to biotic stress, we compared the expression profiles of all RsHSP70 genes in the roots at 0, 7, and 28 dpi with *P*. *brassicae* (Fig. 7). In all, 11 RsHSP70 genes were either up- or downregulated and showed significant differential expression in response to* P*. *brassicae* infection between the clubroot-resistant (RB) and clubroot-susceptible (SB) lines. Only *RsHSP70–5*, *RsHSP70–23*, and *RsHSP70–33* tended to show continuous upregulation at all time points examined. It is noteworthy that the level of RsHSP70–23 expression was 6.83-fold higher in RB than SB roots at 28 dpi (Fig. 7). qRT-PCR analysis showed that the level of *RsHSP70–23* transcript increased sharply in the clubroot-resistant strain (WR1150) compared to the clubroot-susceptible strain (1116 T) after 4 dpi, and remained at a high level from 7 to 28 dpi. These results suggest that RsHSP70–23 may participate in the response to *P*. *brassicae* infection in radish.

### Subcellular localization of RsHSP70–23

**Fig. 8:**
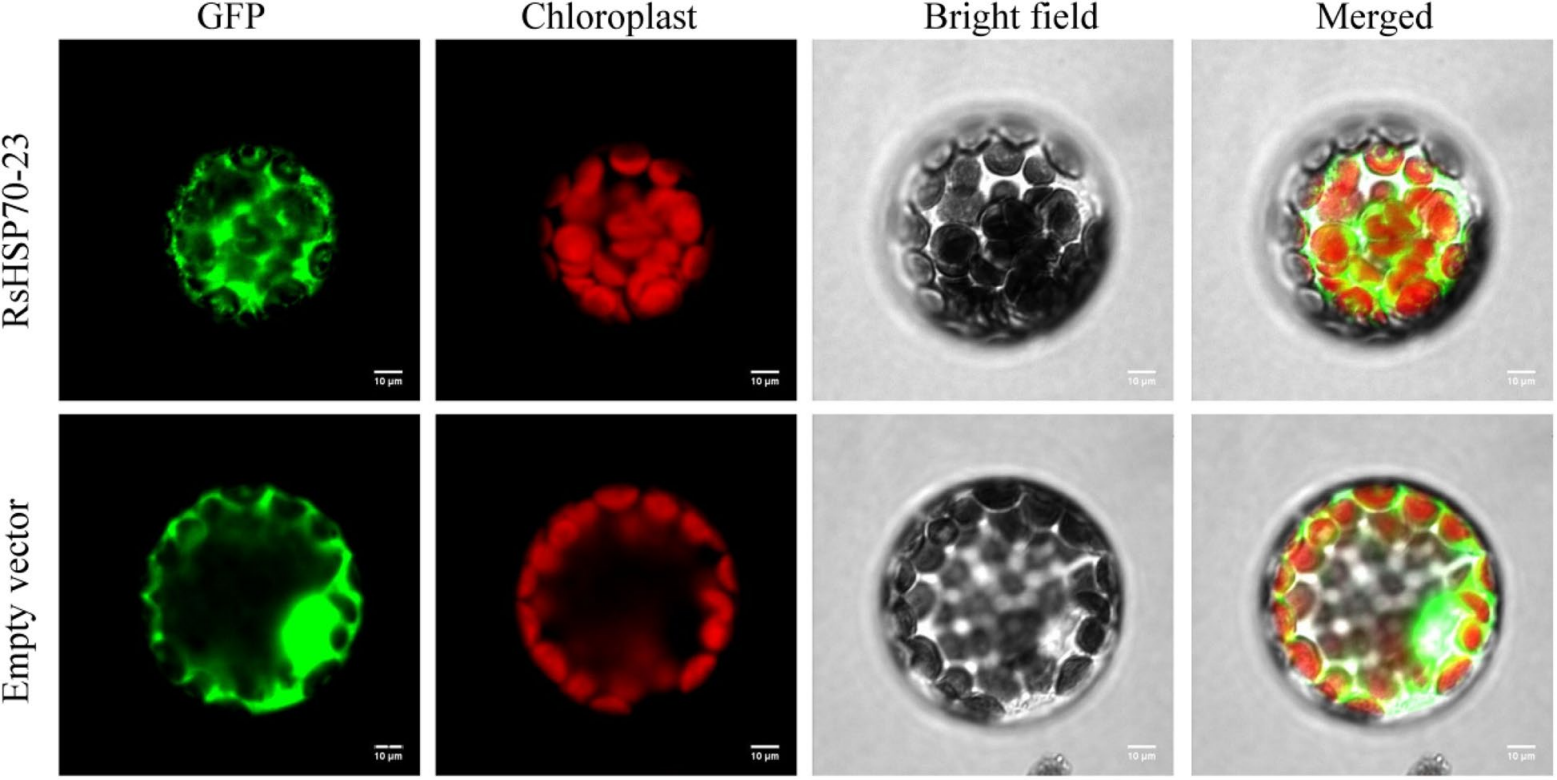
The subcellular localization of RsHSP70-23. Bars = 10 μm

Large-scale synteny analysis revealed that RsHSP70–23 was orthologous to AT5G42020 (BIP2), which is localized to the ER (Fig. 4). WoLF PSORT analysis showed that RsHSP70–23 was also localized to the ER (Table 1), but did not possess the conserved “HDEL” sequence in the C-terminus (Figure S1). To determine the actual subcellular localization of RsHSP70–23 in vivo, GFP-tagged RsHSP70–23 was transiently expressed in *Arabidopsis* protoplasts. Free GFP was evenly distributed in the cytoplasm as expected, and RsHSP70–23-GFP fusion protein was also observed in the cytoplasm (Fig. 8).

### RsHSP70–23 overexpression in response to abiotic stresses in yeast

**Fig. 9:**
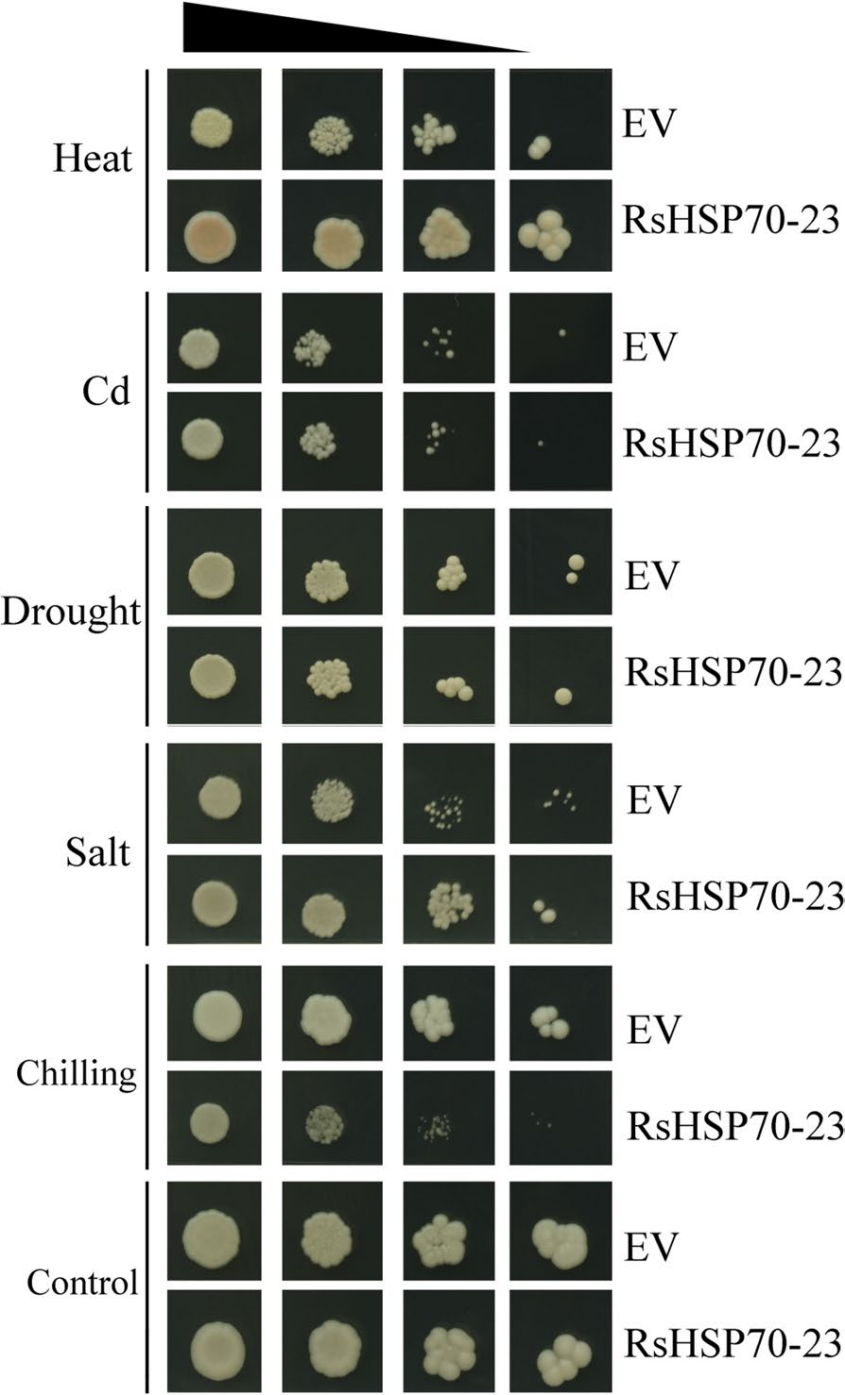
Yeast wild type (EV) strain and RsHSP70-23 overexpressing cell were exposed to abiotic stresses (75 µM-Cd, 1 M-NaCl, 2 M-Mannitol, and cold and heat stresses. Triangles represent the tenfold serial dilutions (the starting OD_600_ is 0.1)

**Fig. 10:**
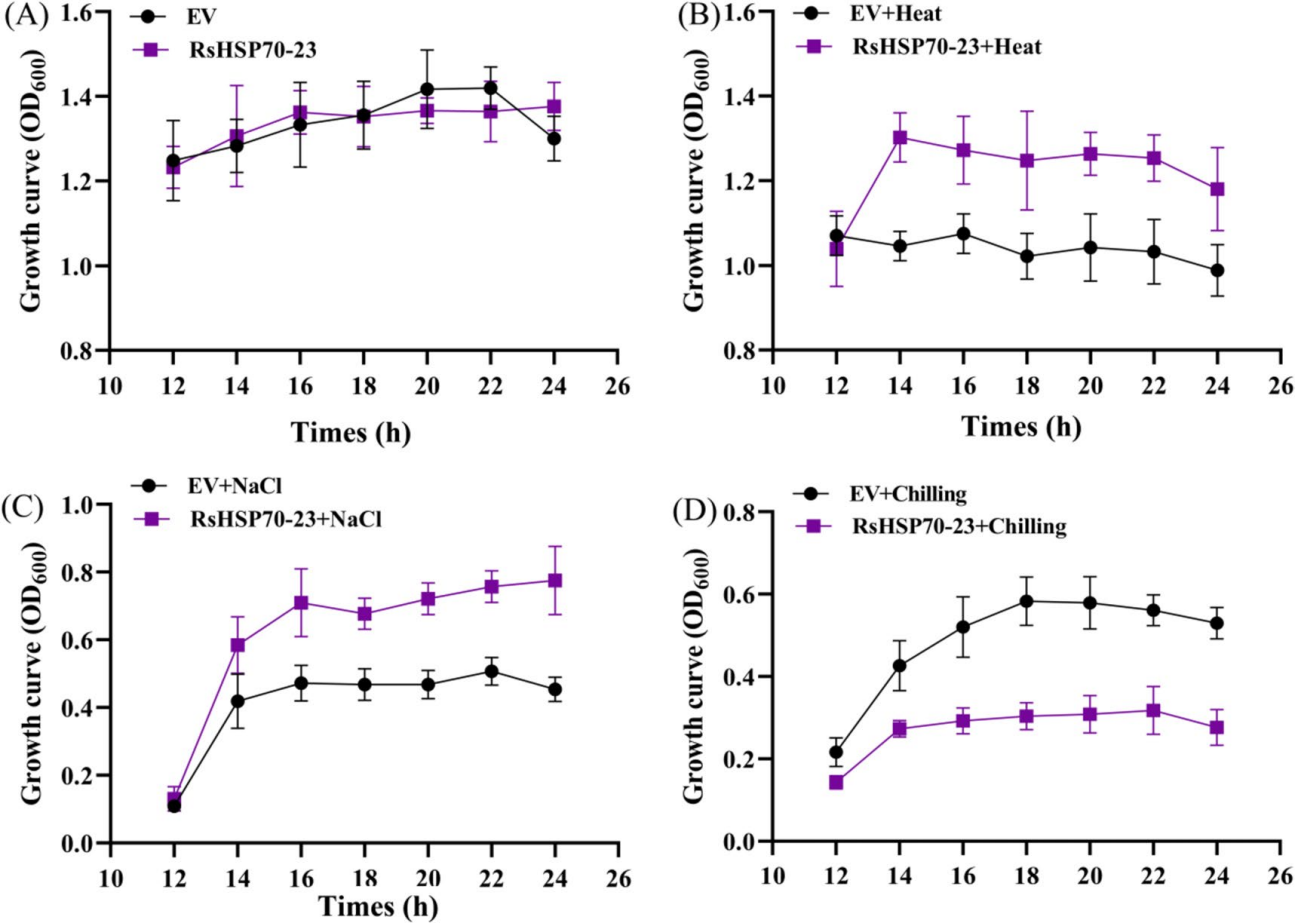
Growth curves of the *RsHSP70-23* gene overexpressing yeast cells and EV (empty vector (yeast WT)) under normal, heat, salt and chilling stress. Cell density was monitored after 12, 14, 16, 18, 20, 22 and 24 h after the treatment. The error bar represents the deviation of three independent replications

To examine whether expression of *RsHSP70–23* responds to abiotic stress, *RsHSP70–23*-overexpressing yeast cells were exposed to 75 µM Cd, 1 M NaCl, 2 M mannitol, chilling (4 °C), and heat (37 °C) stresses (Fig. 9). There were no differences between cells carrying the *RsHSP70–23* overexpression vector or empty vector (EV) control under conditions of mannitol or Cd stress. However, the *RsHSP70–23*-overexpressing cells were sensitive to cold stress compared to EV. Under conditions of NaCl and heat stress, all cells overexpressing *RsHSP70–23* grew faster than those carrying EV. In addition, we conducted growth curves of the yeast cells under heat, chilling and salt stresses (Fig. 10). The growth rate of the *RsHSP70–23*-overexpressing cells showed no difference compared with that of EV under normal conditions, and under salt and heat stress, the optical density was higher than that of EV, whereas under chilling stress, the optical density was decreased compared with that of EV. These results indicated that overexpression of *RsHSP70-23* can alleviate the adverse effects of high temperature and salinity stress on yeast cell growth.

## Discussion

HSP70 proteins have been characterized as molecular chaperones expressed in a variety of plants, and most HSP70 family genes play important roles in the regulation of plant growth, development, and defense [16]. In the present study, 34 potential HSP70 genes were identified based on the *R. sativus* genome sequence, and were divided into six major subfamilies (Fig. 1 and Table 1). Within the same subfamilies, the most closely related RsHSP70 members shared more similarity in terms of motif composition, exon–intron organization, and the corresponding cellular compartments (Fig. 2). Notably, the number of HSP70 members varied among the species tested regardless of genome size and chromosome number (Fig. 1). Radish has twice as many HSP70s as Arabidopsis, which is inconsistent with a whole genome triplication (WGT) event that occurred in the Brassicaceae family [48], indicating that some RsHSP70 genes were lost after expansion. Segmental duplication was the main mechanism of RsHSP70 gene expansion, accounting for 32.35% of cases (Fig. 3). Segmental duplication occurred 2.49–7.58 Mya (Table 2), indicating that the duplication event of all pairs occurred after the divergence of *Arabidopsis* and *Brassica* at about 14.5–20.4 Mya [48], and also after divergence from cabbage and Chinese cabbage at approximately 7.1–10.4 Mya [49]. In addition, approximately 57.15% of segmentally duplicated genes had a Ka/Ks value < 0.1 (Table 2), indicating strong purifying selection, and these gene pairs may have become conserved and their functions tended to be constrained [31].

After duplication, genes may be maintained through subfunctionalization and/or neofunctionalization at the expression or sequence level. Alternatively, duplicated copies may accumulate deleterious mutations and become nonfunctional pseudogenes [50]. In this study, we found some sister pairs with changes in their exon–intron structures and numbers; *RsHSP70–27* contained 2 exons, while its paralogs *RsHSP70–4* and *RsHSP70–11* had 11 and 4 exons, respectively (Fig. 2), indicating that exons were lost during evolution, similar to reports in potato and soybean [12, 51]. Large-scale synteny analysis showed that *AT4G24280* had three orthologous genes in radish (*RsHSP70–4*, *RsHSP70–11*, and *RsHSP70–27*) (Fig. 4). *RsHSP70–27* was not expressed in any of the tissues and/or stress conditions examined, suggesting that it is undergoing pseudogenization. However, *RsHSP70–4* and *RsHSP70–11* exhibited distinct expression patterns between both tissues and various abiotic stress conditions, indicating neofunctionalization (Figs. 5 and 6 and Tables S 5 and S 6). Moreover, there were 13 common syntenic genes between radish, Chinese cabbage, and *Arabidopsis*, thus providing a valuable reference for further understanding of the biological functions of these homologous genes in radish.

HSP70 genes are key components in plant development and in responses to a wide range of abiotic stresses [16]. We found that most *RsHSP70s* exhibited diverse expression profiles in different tissues, particularly in the 20-day leaves and 20-day roots of radish plants (Fig. 5 and Table S 5), suggesting that HSP70 family genes may play important roles in radish seedling development [47]. Under conditions of abiotic stress, transcription factors bind to the *cis*-regulatory elements of stress-responsive gene promoters and specifically initiate transcription of the corresponding genes [51]. In potato, most *StHSP70s* respond to various abiotic stresses (salt, drought, heat, and cold) and hormone treatments (ABA, IAA, GA3, and SA) [12]. More than half of the *HSP70* genes are responsive to ABA, drought, and salt stresses in rice, *Arabidopsis*, and moss [52]. ABA (ABRE), MeJA (CGTCA-motif and TGACG-motif), SA (TCA element), drought (MBS), cold (LTR), and heat (HSE) response elements have been observed in the promoter regions of *PyyHSP70* genes [53]. Here, a variety of hormone and stress response elements were found in the promoter regions of RsHSP70 family genes (Table S 7), where the numbers of HSEs were significantly greater than those of other elements (Table S 8). The two main subunits (5′NGAAN3′ and 5′NTTCN3′) of HSE are recognized by HSF1 [54]. As molecular chaperones, the most important biological function of HSP70s is related to acquired thermotolerance under heat stress, and their expression functions as a negative feedback regulator of heat shock transcription factor (HSF) activity [4–7]. Among the five stress conditions examined here, heat stress induced the greatest number of HSP70 genes in radish (Fig. 6A). These findings suggest that the RsHSP70 genes may respond to multiple hormones and abiotic stresses, particularly heat stress, in radish plants.

Cytosolic and ER-resident HSP70s also play essential regulatory roles in the innate immune response in plant cells [17]. AtBIP2 is localized to the ER and upregulated in response to *Sclerotinia sclerotiorum*,* P*. *syringae*, and FLG-22. In phylogenetic analysis, RsHSP70–23 clustered with AtBIP2 of Group B (Fig. 1), and large-scale synteny analysis also showed that RsHSP70–23 was orthologous with AtBIP2 (Fig. 4 and Table S 4). The expression levels of *RsHSP70–23* in response to *P*. *brassicae* infection were significantly higher in clubroot-resistant than clubroot-susceptible lines (Fig. 7). In addition, BiP genes are induced by multiple abiotic stressors [17, 52]. We found that *RsHSP70–23* showed a lower rate of expression in all tissues examined (Fig. 5) but was significantly upregulated under conditions of salt and heat stress (Figs. 6, 9 and 10). Surprisingly, analysis of the subcellular localization of RsHSP70–23-GFP fusion protein indicated exclusive cytoplasmic localization in *Arabidopsis* protoplasts (Fig. 8). These results suggest that the functions of RsHSP70–23 proteins may have been conserved after the divergence of radish and *Arabidopsis* but also exhibit unique functions through changes in localization during adaptation to changes in the environment.

## Conclusions

In summary, 34 RsHSP70 genes were identified in the radish genome. Their physiochemical properties, phylogenetic relationships, gene organization, gene structures, chromosome distribution, and gene duplication were analyzed, and their expression patterns were characterized to understand their critical functions. These genes may play crucial roles in the growth, development, and stress responses of radish. In addition, RsHSP70–23 was localized to the cytoplasm and was involved in responses to certain abiotic stressors and *P*. *brassicae* infection. This comprehensive characterization of the RsHSP70 gene family will facilitate analysis of HSP70-gene mediated molecular mechanisms of stress responses in root vegetable crops.

### Supplementary Information

**Additional file 1:** **Figure S1.** Multiple sequence alignment of HSP70 proteins.

**Additional file 2:** **Table S1.** The accession number of RNAseq data.

**Additional file 3:** **Table S2.** Primers and corresponding sequences used in the study.

**Additional file 4:** **Table S3.** The MEME motif sequences and lengths of HSP70 gene family proteins in radish.

**Additional file 5:** **Table S4.** The orthologous and paralogous gene pairs of HSP70 proteins among the radish, *Arabidopsis* and Chinese cabbage.

**Additional file 6:** **Table S5.** The TPM expression values of RsHSP70 genes in various tissues.

**Additional file 7:** **Table S6.** Significantly-regulated DEGs at all the stress.

**Additional file 8:** **Table S7.** Summary of abiotic stresses inducible cis-elements is in the promoter regions of HSP70 family genes in radish.

**Additional file 9:** **Table S8.** The detailed information related to the HSF1-binding motif provided by PlantPAN3 online tool.

**Additional file 10:** **Table S9.** Significantly-regulated DEGs under P. brassicae infection.

## Data Availability

All the data obtained in the current study have been presented in this article.
