# Supplementary material for: Systematic analysis of Heat Shock Protein 70 (HSP70) gene family in radish and potential roles in stress tolerance
Source: BMC Plant Biol. 2024 Jan 2;24:2. doi: 10.1186/s12870-023-04653-6 (PMC10759535; doi:10.1186/s12870-023-04653-6)
Supplement: Supplementary file 3 — Additional file 3: Table S2. Primers and corresponding sequences used in the study. [file 12870_2023_4653_MOESM3_ESM.doc]

Table S3. The MEME motif sequences and lengths of HSP70 gene family proteins in radish.

| Motif | Width | Best possible match |
| --- | --- | --- |
| 1 | 100 | MTTLIPRNTTIPTKKEQVFSTYSDNQPGVLIQVYZGERARTKDNNLLGKFELSGIPPAPRGVPQIEVCFDIDANGILNVSAEDKTTGKKNKITITNDKGR |
| 2 | 98 | VFDLGGGTFDVSVLTIEEGVFEVKATAGDTHLGGEDFDNRLVBHFVQEFKRKSKKDJSKBPRALRRLRTACERAKRTLSSTAQTTIEJDSLFEGIDFY |
| 3 | 57 | LTRARFEELNMDLFRKCMEPVEKCLRDAKLDKSDVHEVVLVGGSTRIPKVQZLLQDF |
| 4 | 100 | IEKMVQEAEKYKSEDEEHKKKVEAKNALENYAYNMRNTIRDEKIGEKLPAADKKKIEDSVEEAIZWLDGNQTAEADEFEDKMKELESVCNPIIAKMYQGA |
| 5 | 52 | DAVVTVPAYFNDAQRQATKDAGRIAGLBVLRIINEPTAAAJAYGJDKKGGET |
| 6 | 36 | NGKEPCKSINPDEAVAYGAAVQGAILSGEGNEKVLD |
| 7 | 36 | KDEIEKMVKEAERFAKEDKEKKDAIDTKNAAESYVY |
| 8 | 36 | AAKRQAVVNPENTVFDVKRLIGRRFDDPEVQKDMKM |
| 9 | 43 | VIGIDLGTTNSCVAVMEGGKPEIIENAEGNRTTPSVVAFTKSG |
| 10 | 36 | ASYKGEGKQFSPEZISAMVLTKMKETAEAYLGKKVK |
| 11 | 50 | GPAIGIDLGTTYSCVGVWQHDRVEIIANDQGNRTTPSYVAFTDTERLIGD |
| 12 | 14 | DVTPLSLGJETAGG |
| 13 | 70 | VHESFPFSISLAWKGAAVDAQNGGAENQQSTIVFPKGNSIPSVKALTFYRSGTFSVDVQYIDVTDLQAPP |
| 14 | 68 | QTEKQLKELGEKIPGEVKEEIEAKLQELKDKIASGSTQEIKDTIAALNQEVMQIGZSMYGQPGAGAGG |
| 15 | 19 | DDEAPPSSGGAGPKIEEVD |
| 16 | 99 | LRYKELTERPVVIGYLGKYLTEYKEAAKEWDSKFDWJPKEEKQEVLKEAEEVEAWLEEKZQZQEKLPKWAKPVFLSDDVYAKVFALDDFVRPINRKPKP |
| 17 | 47 | EKEKIEAATKEALEWLDENQNSEKEEYEEKLKEVEAVCNPIITAVYQ |
| 18 | 26 | MRIINEPTAAAIAYGLDKKATSVGEK |
| 19 | 69 | QYPISGLIDETEKRKSPNLVAPHKGDRHFGLERAGILARDPGDVYIZIRDWVGKPFKNVKDFNDSVYLP |
| 20 | 100 | RHVIFYDMGSSSTYAALVYYSAYNEKEFGKNVSVNQFQVKDVRWDSGLGGQSMEMRLVEYFADEFNKQLGNGVDVRKFPKAMAKLKKQVKRTKEILSANT |
